# Neurotype-Matching, but Not Being Autistic, Influences Self and Observer Ratings of Interpersonal Rapport

**DOI:** 10.3389/fpsyg.2020.586171

**Published:** 2020-10-23

**Authors:** Catherine J. Crompton, Martha Sharp, Harriet Axbey, Sue Fletcher-Watson, Emma G. Flynn, Danielle Ropar

**Affiliations:** ^1^Patrick Wild Centre, Division of Psychiatry, Centre for Clinical Brain Sciences, University of Edinburgh, Edinburgh, United Kingdom; ^2^School of Psychology, University of Nottingham, Nottingham, United Kingdom; ^3^School of Education, Durham University, Durham, United Kingdom; ^4^Salvesen Mindroom Research Centre, University of Edinburgh, Edinburgh, United Kingdom; ^5^School of Psychology, Queen’s University, Belfast, United Kingdom

**Keywords:** autism, Double Empathy Theory, rapport, interaction, communication, neurodiversity

## Abstract

The Double Empathy Problem suggests that communicative difficulties between autistic and non-autistic people are due to bi-directional differences in communicative style and a reciprocal lack of understanding. If true, there should be increased similarity in interaction style, resulting in higher rapport during interactions between pairs of the same neurotype. Here, we provide two empirical tests of rapport, with data revealing whether self- and observer- rated rapport varies depending on the match or mismatch in autism status within a pair. An additional opportunity afforded by these data is to examine the effect of the autism status of the rater on the perceived rapport between matched and mismatched pairs. In Study 1 72 participants were allocated to one of three dyad conditions: autistic pairs (*n* = 24), non-autistic pairs (*n* = 24) and mixed pairs (*n* = 12 autistic; *n* = 12 non-autistic). Each participant completed three semi-structured interactions with their partner, rating rapport after each interaction. Non-autistic pairs experienced higher self-rated rapport than mixed and autistic pairs, and autistic pairs experienced higher rapport than mixed pairs. In Study 2 (*n* = 80) autistic and non-autistic observers rated interactional rapport while watching videoed interactions between autistic pairs, non-autistic pairs, and mixed pairs (*n* = 18, a subset of participants in Study 1). Mixed pairs were rated significantly lower on rapport than autistic and non-autistic pairs, and autistic pairs were rated more highly for rapport than non-autistic pairs. Both autistic and non-autistic observers show similar patterns in how they rate the rapport of autistic, non-autistic, and mixed pairs. In summary, autistic people experience high interactional rapport when interacting with other autistic people, and this is also detected by external observers. Rather than autistic people experiencing low rapport in all contexts, their rapport ratings are influenced by a mismatch of diagnosis. These findings suggest that autistic people possess a distinct mode of social interaction style, rather than demonstrating social skills deficits. These data are considered in terms of their implications for psychological theories of autism, as well as practical impact on educational and clinical practice.

## Introduction

Rapport is defined by mutually experienced co-ordination, positivity, and attentiveness within a social interaction (Tickle-Degnen and Rosenthal, 1990). It is marked by a harmony and affinity between two people (Bernieri, 2014), and is a key component in constructing and developing successful interpersonal interactions (Cappella, 1990). As rapport relates to the quality of a relationship between two people, it is distinct from many other psychological constructs which are situated within individuals, rather than within interactions (Bernieri, 2014). Feelings of rapport can be influenced by social context with individuals from the same or similar social groups reporting higher rapport (Miles et al., 2011), even when those groups are defined by arbitrary or minimal criteria (Tajfel, 1981; Macrae and Bodenhausen, 2000; Brewer, 2007). Non-verbal and verbal communicative behaviors, including facial expressions, eye contact, postural mirroring, and tone play an important role in building rapport in people presumed to be neurotypical (Tickle-Degnen and Rosenthal, 1990); while not exhibiting these behaviors is related to poorer rapport (Richmond and McCroskey, 1995; Grahe and Bernieri, 1999; Hove and Risen, 2009). As difficulties with processing and expressing verbal and non-verbal social cues amongst autistic individuals have been well documented (Bottema-Beutel et al., 2019; Sasson et al., 2020), we might expect this to subsequently impact upon their development of rapport with others.

Autism is typically characterized by differences in social communication and interaction (American Psychiatric Association, 2013) compared with neurotypical norms. Popular attempts to explain autism, such as accounts like theory of mind (Frith, 2001), executive functioning (Ozonoff et al., 1991), or social motivation (Chevallier et al., 2012) adopt a deficit-based model. For instance, theory of mind explanations propose that social difficulties arise from a cognitive deficit residing in the autistic person preventing them from being able to infer, understand, or predict the behavior and intentions of others (Baron-Cohen et al., 1985; Gernsbacher and Yergeau, 2019). Experimental research showing that autistic people are unable to attribute mental states to others is believed to underlie autistic difficulties in social communication (Frith, 2001). Specifically, theory of mind deficits in autistic individuals has been linked to difficulties in identifying facial expressions (Uljarevic and Hamilton, 2013), and tone of voice (Rutherford et al., 2002). Additionally, autistic people have differences in frequency and patterns of eye contact, and postural and behavioral mirroring (Senju and Johnson, 2009; Hamilton and Marsh, 2013). Given these behaviors are thought to be related to rapport, it would be expected that interactions with and between autistic people would elicit low rapport. Applying a deficit model framework to paired interactions, autistic people should have the same difficulties interacting with autistic and non-autistic people (due to impairments in social communication) but difficulties would be compounded when two autistic people interact. A hypothesis based on this framework would predict that rapport between two non-autistic people would be highest, rapport between two autistic people would be lowest, and rapport between an autistic person and a non-autistic person would sit between these extremes.

Until recently, approaches to studying autism have been framed by neurotypical definitions of being social (Heasman and Gillespie, 2019a) and yet those with autism have a divergent neurotype, which often makes their mode of social communication different (Kapp et al., 2013). Increasingly, deficit-based paradigms are challenged by ideas grounded in the social model of disability, which proposes that autistic difficulties emerge as a result of systemic barriers in society (Kapp et al., 2013). There is increasing evidence suggesting that non-autistic people contribute to difficulties in interactions between autistic and non-autistic people (e.g., Edey et al., 2016; Sheppard et al., 2016; Sasson et al., 2017; Heasman and Gillespie, 2019a; Crompton et al., 2020a, b; Keating and Cook, 2020). This phenomenon has been conceptualized through the Double Empathy Problem, a theory which suggests that communicative difficulties between autistic and non-autistic people are due to bi-directional differences in communicative style and a reciprocal lack of understanding (Milton, 2012; Milton et al., 2018). The Double Empathy Problem contrasts with more traditional models of interaction in autism (Frith, 1994; Chevallier et al., 2012) and the diagnostic criteria (American Psychiatric Association, 2013; World Health Organization, 2020), which emphasize pervasive deficits in social interaction that are inherent in autistic populations. Instead, it suggests that difficulties arise due to a mismatch between autistic and non-autistic interaction styles, resulting in a decrement in social understanding on both sides.

Empirical support for the Double Empathy Problem is based on two strands of research. One area of research has explored non-autistic people’s difficulties in interacting with autistic people. Non-autistic people are less accurate at deciphering the facial expression of autistic people (Sheppard et al., 2016) and struggle to interpret autistic people’s mental states (Edey et al., 2016). Struggling to read autistic social cues is related to non-autistic people liking autistic people less (Alkhaldi et al., 2019), and non-autistic people are less willing to interact with autistic people (Sasson et al., 2017). Non-autistic people are also less likely to want to spend time or interact with autistic people than with non-autistic people (Morrison et al., 2020). These biases against autistic individuals are formed quickly by non-autistic people, and do not change with increased exposure (Sasson et al., 2017). Non-autistic people overestimate how egocentric autistic family members are (Heasman and Gillespie, 2018), while also overestimating the helpfulness of their own behaviors toward autistic people (Heasman and Gillespie, 2019b). Taken together, this body of research provides evidence that autistic social difficulties may be in part due to the perceptions of, and judgments made by, non-autistic people.

The second research focus has been to examine inter-autistic communication and interaction. There are distinctive features of interactions between autistic people (Heasman and Gillespie, 2019a; Granieri et al., 2020), and autistic people qualitatively report that their interactions with other autistic people are more comfortable and easier compared with interactions with non-autistic people (Crompton et al., 2020a). Though autistic people may perceive other autistic people as being more awkward, less attractive, and less socially warm than non-autistic people, autistic people still express interest in future interactions with other autistic people (DeBrabander et al., 2019; Morrison et al., 2020), suggesting that autistic people base their social judgments on fundamentally different criteria to non-autistic people. Indeed, autistic people are less likely to find non-typical social behaviors in other autistic people problematic (Sng et al., 2020). Autistic people disclose more personal information to other autistic people, feel close to other autistic people (Morrison et al., 2020), empathize more with autistic people, and are more motivated to help them than non-autistic people (Komeda et al., 2019). While little is known about the mechanisms that underlie comfortable interactions between autistic people, autism-specific communication styles are associated with more positive first impressions by other autistic people (Granieri et al., 2020).

The Double Empathy Problem suggests that difficulties in interaction occur due to a lack of reciprocity between different neurotypes, and proposes that there will be increased reciprocity, and therefore higher rapport, between people of the same neurotype. According to the Double Empathy Problem, it would be hypothesized that rapport between autistic pairs and non-autistic pairs would be better than rapport within mixed autistic and non-autistic pairs.

Rapport has been measured using combinations of associated characteristics, such as warmth, empathy, understanding, friendliness and genuineness between those in the interaction (Tickle-Degnen and Rosenthal, 1990). Studies of rapport in dyadic interactions typically examine either self-rated questionnaires (i.e., each participant in the interaction rates the rapport they felt in their interaction, e.g., Frisby and Martin, 2010), or observer-rated questionnaires (i.e., observers watch video clips of dyads interacting, and rate the rapport between the two participants, e.g., Hall et al., 2009). While self-rated rapport can give an indication of one’s personal experience of a social interaction, this judgment may be prone to biases (Pronin et al., 2004). Observer-ratings however may allow for a complementary, and more objective assessment of interpersonal interaction rapport between pairs of individuals.

In this paper we aim to contrast the deficit model framework with the Double Empathy Problem by conducting two studies assessing rapport between pairs of autistic adults, pairs of non-autistic adults, and mixed pairs where one person was autistic and one was non-autistic. Study 1 included self-rated rapport, as experienced during task-based dyadic interactions where each person’s diagnosis status (autistic or non-autistic) was known by the other. Study 2 involved autistic and non-autistic observers rating rapport for videoed informal interactions between autistic pairs, non-autistic pairs, and mixed pairs. In this study the observers were blind to the diagnostic status of the participants engaging in social interaction within the videos. If social interaction difficulties experienced by autistic individuals were due to a mismatch in communication style, as posed by the Double Empathy Problem, we would expect the lowest ratings of rapport in mixed pairs in Studies 1 and 2. If however, rapport ratings were lowest in the autistic dyads (in both studies) these findings may align better with a deficit framework. A further component of both studies is the inclusion of autistic and non-autistic raters in each, which allowed us to explore whether rapport is judged similarly (both for self and others) within these two populations. If autistic individuals fail to pick up on appropriate social cues during or while viewing a social interaction, we would expect their judgments of rapport to differ from non-autistic individuals.

## Study 1: Self-Rated Rapport in Autistic, Non-Autistic, and Mixed Pairs

### Ethics and Recruitment

This study was carried out in accordance with the British Psychological Society’s Code on Human Research Ethics. Experimental procedures for Study 1 were reviewed and approved by the University of Edinburgh Research Ethics Committee. All participants provided written informed consent prior to participating. Participants were recruited through community networks, social media, and local autism organizations.

### Participants

Seventy-two adults participated: twenty-four adults in each of the autistic, non-autistic, and mixed groups. The mixed group therefore included 12 autistic and 12 non-autistic participants. A prospective power analysis was run, indicating 95% power to detect a medium effect of 0.5 at the standard 0.05 alpha error probability with a sample size of 66. The three groups were matched on age, gender, years of education, and IQ (Table 1). All spoke English to a native level and did not have a clinical diagnosis of social anxiety disorder. Participants also completed the Wechsler Abbreviated Scale of Intelligence II (WASI-II) (Wechsler, 2011), a measure of IQ, with all participants scoring within a typical range. Demographics are presented below based on dyad types for the purposes of the study, and demographic data based on the individual data (autistic, and non-autistic participants, *n* = 36 in each group) are shown in Supplementary Material 1 for additional context.

**TABLE 1:** Descriptive statistics and group comparisons [Mean (Standard Deviation)] for Study 1 participants on demographic variables, IQ, and autistic traits.

	Non-autistic (*n* = 24)	Autistic (*n* = 24)	Mixed (*n* = 24)	Comparisons
Age	37.92 (14.39)	37.33(13.13)	35.25 (10.76)	X^2^(2) = 0.27, *p* = 0.87
Gender	21F, 3M	18F, 3M, 3NB^*b*^	18F, 6M	Fisher’s exact test *p* = 0.17
Years of Education	17.83 (1.52)	17.44 (2.80)	17.12 (1.98)	X^2^(2) = 1.83, *p* = 0.40
IQ – WASI-II^*a*^	115.04 (11.78)	114.42 (16.89)	117.79 (13.62)	*F*(2,69) = 0.38, *p* = 0.68
Autism Quotient	13.21 (5.44)	35.58 (6.18)	26.88 (14.27)	*X*^2^(2) = 32.26, *p* = 0.001
Age of Diagnosis	NA	30.55 (12.72)	30.89 (10.20)	*X*^2^(1) = 0.36, *p* = 0.85

*^*a*^Wechsler Abbreviate Scale of Intelligence -II. ^*b*^Non-binary.*

Thirty-three autistic participants reported having received a diagnosis by a clinician. An additional three participants self-identified as autistic. Participants who self-identified as autistic also scored above 32 on the Autism Quotient (AQ) (Baron-Cohen et al., 2001) and above 72 on the Ritvo Autism-Aspergers Diagnostic Scale-Revised (Ritvo et al., 2011) indicating not only high levels of autistic traits but also a self-rating above a diagnostic threshold. All non-autistic participants scored below 32 on the AQ, indicating low levels of autistic traits (Baron-Cohen et al., 2001).

### Materials and Procedure

All participants took part in three experimental tasks using a diffusion chain method (Crompton et al., 2020b). This procedure involves a series of dyadic interactions in which an individual first observes a researcher complete a task, and then completed that task with a second participant. The second participant then completed the task with a third participant, and so on, until an eighth participant completes the task. In effect this allowed for 7 dyadic interactions between participants per chain (and thus yielding 63 interactions in total; 21 autistic, 21 non-autistic, and 21 mixed interactions). Only two participants were in the same room, and interacting, at any one time. Each chain of eight participants attended a different research day, hosted at the University of Edinburgh Division of Psychiatry.

Before the study commenced, participants were aware whether they were in an autistic, non-autistic, or mixed dyad. Participants did not meet before the first task started, and were isolated in separate rooms whilst they waited for their turn to take part in the study. The first dyadic task involved building a tower out of spaghetti and plasticine (Caldwell and Millen, 2008), the second involved sharing a fictional story (see Crompton et al., 2020b), and the third involved participants creating geometric animal shapes from a Rubiks Twist (^TM^). Each task took between 1 and 5 min, and participants interacted with each other freely while completing each task.

After each task, participants indicated their feelings of rapport using a 100-point scale with five dimensions: ease, enjoyment, success, friendliness, and awkwardness (reverse scored). Participants indicated a score for each dimension by drawing a cross on a horizontal line, indicating a scale from 1 to 100. The five dimensions had a Cronbach’s alpha of 0.93, and so were summed to create a single scale of interactional rapport for use in subsequent analyses.

### Design

This study used a between-groups design, comparing self-rated rapport in autistic, non-autistic, and mixed groups.

### Results

For each dyad, a pair mean rapport score was calculated to reflect the overall rapport experienced by both participants in each dyadic interaction. This was calculated as the average of the rapport scores of both participants within each pair for each task. There was no significant interaction between the three dyadic tasks and the three groups (see Supplementary Figure 1), and so a summed mean was used in subsequent analyses, calculated as the mean of the pair’s mean rapport scores for each of the three tasks (minimum = 0, maximum = 500).

**FIGURE 1:**
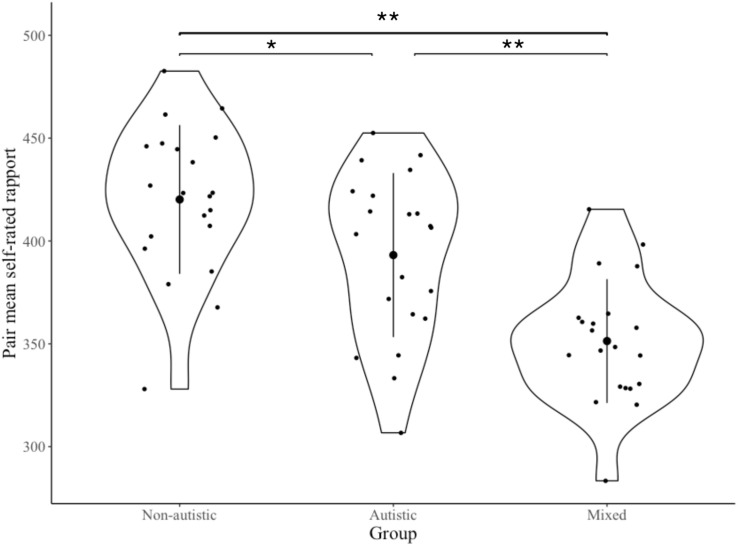
Mean Pair self-rated rapport for non-autistic, autistic, and mixed groups. Bold dot indicates mean, line indicates standard deviation, and violin plot showing distribution of the data, *p < 0.05, **p < 0.001.

The summed pair mean rapport scores met assumptions of normality and homogeneity of variance, and a subsequent one-way ANOVA found a significant difference in overall rapport between the three groups [*F*(2,60) = 19.89, *p* < 0.001. *Post hoc* comparisons using Tukey’s HSD indicated that the non-autistic group experienced higher self-rated rapport than the mixed (*p* < 0.000001) and autistic group (*p* < 0.05), and the autistic group experience higher self-rated rapport than the mixed group (*p* < 0.001) see Figure 1].

**FIGURE 2:**
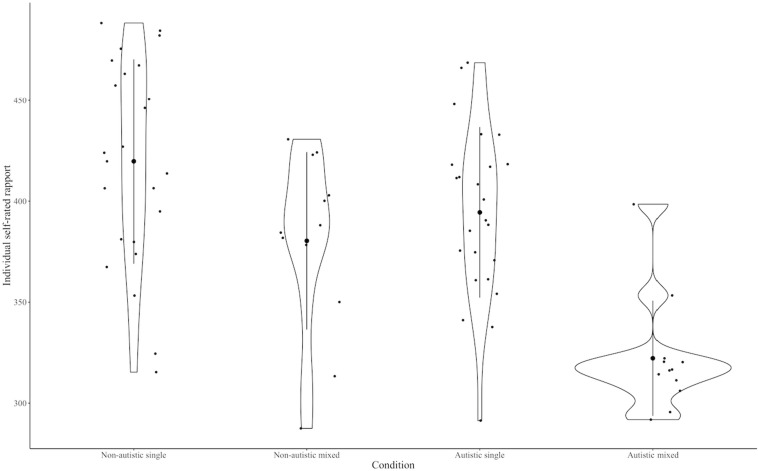
Self-rated rapport for autistic and non-autistic participants in single and mixed social contexts. Bold dot indicates mean, line indicates standard deviation, and violin plot showing distribution of the data.

Subsequent analysis explored potential effects of the participant’s neurotype (autistic or non-autistic) and the social context (i.e., whether participants were in a matched chain with participants of the same neurotype, or a mixed chain with participants from a different neurotype) on self-rated rapport (Figure 2). A two-way ANOVA showed an effect of neurotype, with lower ratings of rapport in the autistic group [autistic mean = 370.38, non-autistic mean = 406.62, *F*(1,68) = 12.32, *p* < 0.001], and an effect of social context, with lower ratings in the mixed group [mixed mean = 351.30, matched mean = 407.1, *F*(1,68) = 25.97, *p* < 0.001]. However, there was no significant interaction between rater neurotype and social context [*F*(1,68) = 2.25, *p* = 0.13].

### Summary

This study examined how autistic and non-autistic people self-rated rapport with autistic and non-autistic partners. Participants completed short tasks with a partner, and afterward rated their experiences of rapport on a 5-dimensional scale.

Results showed that non-autistic pairs experienced higher self-rated rapport than autistic pairs, and both autistic and non-autistic pairs, and mixed pairs experienced lower rapport than both autistic pairs and non-autistic pairs. Regardless of individual neurotype, rapport is lower within mixed pairs compared with single neurotype pairs.

Additionally, examining the effect of the social context (i.e., whether in a matched or mixed-neurotype pair), showed that both autistic and non-autistic participants experienced lower rapport in mixed pairs. A lack of interaction with rater neurotype indicates that the lower rapport experienced in the mixed pairs is not driven by participants of a particular neurotype: both autistic and non-autistic participants had lower rapport within mixed pairs than in single neurotype pairs. However, given the small number of participants in each group when analyzing the data in this way (*n* = 12 each of autistic and non-autistic people in the mixed group), low statistical power may have contributed to the lack of a significant effect.

## Study 2: Observer Rated Rapport of Autistic, Non-Autistic, and Mixed Pairs

### Ethics and Recruitment

This study was carried out in accordance with the British Psychological Society’s Code on Human Research Ethics. Experimental procedures were reviewed and approved by the University of Edinburgh Psychology Research Ethics Committee, the University of Nottingham (Psychology) Research Ethics Committee, and the University of Durham (Education) Research Committee. All participants provided written informed consent prior to participating. Participants were recruited through community networks, social media, and local autism organizations.

### Participants

Study 2 included eighty participants (40 autistic and 40 non-autistic) recruited across three sites: 24 at the University of Edinburgh, 22 at the University of Durham, and 34 at the University of Nottingham. A prospective power analysis was run, indicating 95% power to detect a medium effect of 0.5 at the standard 0.05 alpha error probability with a sample size of 54. Two participants (one autistic and one non-autistic) were excluded after testing, due to having an AQ score which was out of range (i.e., below or above 32 respectively) for their stated neurotype.

The final participant groups (39 autistic and 39 non-autistic individuals) were matched on age, gender and years of education. All spoke English to a native level. All non-autistic participants scored less than 32 on the Autism Quotient, indicating low levels of autistic traits (Baron-Cohen et al., 2001). Autistic participants were either clinically diagnosed (*n* = 36), or self-diagnosed (*n* = 3) and scored above 32 on the Autism Quotient (AQ) (Baron-Cohen et al., 2001). Demographic information for the autistic and non-autistic participants are shown in Table 2.

**TABLE 2:** Descriptive statistics and group comparisons [Mean (Standard Deviation)] for Study 2 participants on demographic variables and autistic traits.

	Non-autistic (*n* = 39)	Autistic (*n* = 39)	Comparisons
Age	33.74 (13.31)	34.31(13.20)	*U* = 1.28, *p* = 0.26
Gender	25F, 14M	23F, 14M, 2NB^*a*^	Fisher’s exact test *p* = 0.56
Years of Education	17.17 (2.26)	17.36 (3.12)	*U* = 0.054, *p* = 0.817
Autism Quotient	15.95(6.27)	37.50 (8.64)	*U* = 48.43, *p* = 0.001
Age of Diagnosis	NA	26.69 (12.77)	NA

*^*a*^Non-binary.*

### Materials and Procedure

#### The Paired Interaction Videos

Nine video stimuli were created for use in Study 2. These videos featured a subset of eighteen participants from Study 1. Videos featured three different pairs of autistic participants, three different pairs of non-autistic participants, and three different pairs of participants where one person was autistic and one was non-autistic (hereafter “mixed” pairs).

Each video featured a 2-min interaction between participant pairs (the first 2 min of a longer interaction, shortened to reduce task demand and length), who sat together at a table with their upper body and heads visible to viewers. Participants in the videos had been given a prompt sheet of paper providing basic statements to frame the interaction, for example “Tell me about where you live.” Participants had not met each other before this interaction took place. After each interaction, participants in the videos completed the Rapport Measure, described in Study 1. Details about the demographics of video participants are outlined in Supplementary Table 2.

#### Observer Ratings of Rapport

Participants (observers) individually watched 3 videos, one from each dyad condition (i.e., autistic, non-autistic, mixed, with the order of presentation counterbalanced between observers). After each video, observers completed ratings of rapport using the same scale used in Study 1, measuring how easy, enjoyable, friendly, successful and awkward they thought the interaction between the observers in the video appeared, on a scale of 0–100. The observers did not know the diagnosis of individual people in the video, however they were aware that one or more people in the videos may have a diagnosis of autism. Observers watched each video start to finish before marking any responses to ensure they had fully seen and processed each interaction. Observers then completed the AQ (Baron-Cohen et al., 2001).

### Design

This study used a mixed design, exploring how neurotype (autistic or non-autistic) affects observer-rated rapport of autistic, non-autistic, and pair dyads interacting in video clips. Researchers were blind to which pair was which in the videos, making it a double-blind study to minimize bias in the results.

### Results

The five dimensions on the rating scale had a Cronbach’s alpha of 0.91, and so were summed to create a single value of interactional rapport for use in subsequent analyses.

Initial review of the data revealed an outlier within the autistic group with lower overall rapport scores on the same neurotype pairings (autistic and non-autistic). A closer look at this individual’s data showed no evidence of misunderstanding the scale (i.e., reversing coding) and as the results remained the same when conducted with the outlier removed it was decided to retain their data. Data in one of the dyad conditions (autistic pairs) were moderately skewed (−0.56) thus did not meet the assumption of normality. Another group (mixed pairs) did not meet the assumption of homogeneity of variances. However, as ANOVA is reported to be robust against small variations in the data distribution (Schmider et al., 2010) it was decided to proceed with parametric analysis.

**FIGURE 3:**
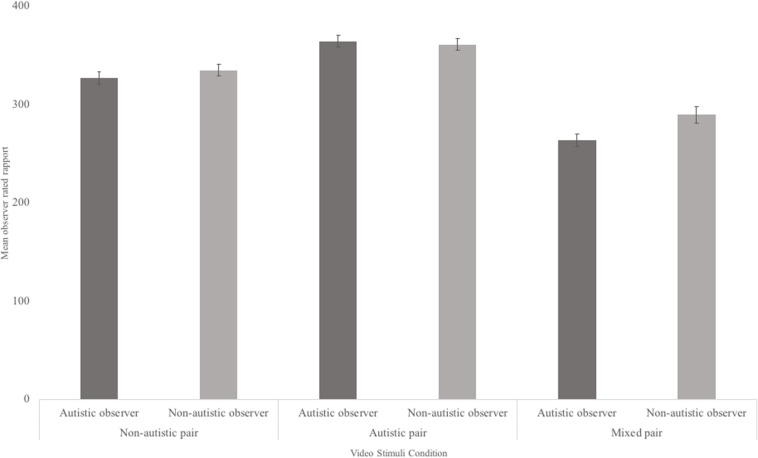
Mean and standard error for observer rated rapport of non-autistic, autistic, and mixed pairs, by rater neurotype.

A mixed 2 × 3 ANOVA was carried out to explore whether there were any group differences in how autistic and non-autistic participants judged rapport of social interactions between autistic, non-autistic, and mixed pairs (Figure 3). Results showed a main effect of dyad condition [*F* = (1.67,127.57) = 24.07, *p* < 0.001; non-autistic mean = 331.99, autistic mean = 364.25, mixed mean = 275.43]. Paired-sample *post hoc* tests revealed significantly lower rapport ratings for mixed pairs than autistic [*t*(77) = −6.43, *p* < 0.001] and non-autistic pairs [*t*(77) = −3.81, *p* < 0.001]. Furthermore, autistic pairs were found to have significantly higher ratings of rapport than non-autistic pairs [*t*(77) = 3.38, *p* = 0.001]. Between subject comparisons showed that both autistic and non-autistic observers did not differ in how they rated rapport in general across all videos [*F*(1,76) = 0.428, *p* = 0.52]. In addition, there was no significant interaction between rater diagnostic status and dyad condition [*F*(2,127.57) = 0.75, *p* = 0.46].

**FIGURE 4:**
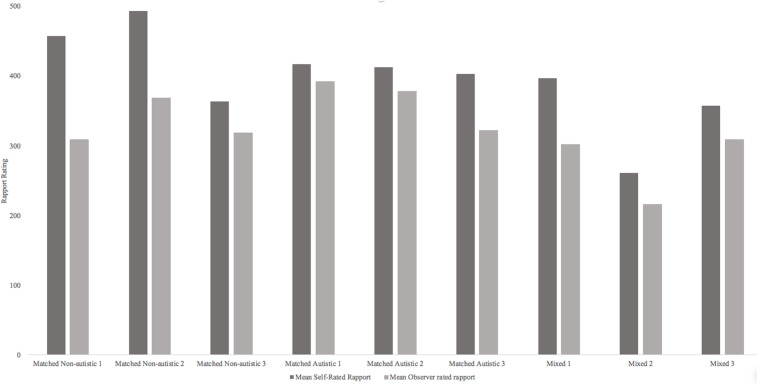
Mean self and observer rated rapport for the nine video stimuli.

Though the small sample size prohibited formal comparison of self- and observer-rated rapport in Study 2, Figure 4 illustrates how participants who created the video stimuli rated their own rapport alongside how observer participants rated their rapport. Though these data are too limited for significance testing, it appears that autistic participants’ self-ratings of rapport in matched autistic pairs are more similar to observer ratings of rapport (mean difference between self and observer rated rapport = 46.91). There is a greater difference between self-rated and observer-rated rapport in matched non-autistic pairs (mean difference between self and observer rated rapport = 106.35).

### Summary

Study 2 examined how observers rated rapport between autistic, non-autistic, and mixed pairs, and whether the diagnostic status of the rater (autistic or non-autistic) affected ratings of rapport in different neurotype pairs. The results indicate that participants, regardless of diagnostic status, give poorer ratings of rapport for mixed neurotype pairs than for matched neurotype pairs. This suggests a mismatch between neurotypes results in lower ratings of rapport, and that subtle verbal and non-verbal cues to rapport are similarly perceptible by autistic and non-autistic individuals. Interestingly, rapport scores were significantly higher for the autistic pairs than non-autistic pairs, indicating that the autistic dyads may display even greater social signals of shared enjoyment and ease when interacting with one another, as viewed by an external observer.

An exploratory comparison between participants’ own judgments of rapport and an observer’s ratings, suggests autistic participants’ self-rating of rapport are more in line with others’ ratings of rapport. There was a greater discrepancy between non-autistic participants’ estimates of their rapport with a partner compared with observers’ rating of the same social interaction.

## Discussion

Studies 1 and 2 examined perceptions of rapport between autistic pairs, non-autistic pairs, and mixed pairs. Though these two studies are not directly comparable as they involved different measures (self or other rated) of different social situations (task focused or informal chat) they both provide evidence that rapport between mixed pairs of individuals is significantly lower than in same neurotype pairs. These findings are consistent with our predictions and offer support for the Double Empathy Problem. A further common finding in both studies is that there were no differences in the pattern of rapport ratings between autistic raters and non-autistic raters. This suggests that autistic individuals discriminate between good and poor rapport between different dyad pairs like non-autistic pairs. In addition to these findings which are common to both studies, the results specific to each study and their implications will be discussed below.

In Study 1 it was demonstrated that self-rated rapport was poorer in mixed pair groups than in same pair (autistic-autistic; non-autistic-non-autistic) groups, as predicted according to the Double Empathy Problem. The results also showed that within the mixed dyad group both autistic and non-autistic people experience lower rapport when interacting with someone of a different neurotype. This provides evidence that the social difficulties autistic individuals experience when interacting with a non-autistic individual may at least partly be attributed to a mismatch in neurotype. Thus, social difficulties for autistic people may be relational in nature, rather than an individual impairment as posited by accounts which adopt a deficit model. These findings are in line with a recent review which argues that there is growing evidence to suggest that a theory of mind explanation for social difficulties in autism is questionable (Gernsbacher and Yergeau, 2019), and echoes findings from other research using a range of methodologies to examine the bi-directional nature of social interaction, considering communication as a joint experience rather than at the individual level (De Jaegher and Di Paolo, 2007; Bottema-Beutel, 2017; Sterponi and De Kirby, 2017). If rapport is constructed from subtle verbal and non-verbal cues during social interactions, then autistic individuals must be sufficiently able to detect these to discriminate between the mixed neurotype and same neurotype groups.

More broadly, these findings fit with the wider psychological literature on in-group/out-group effects (e.g., Tajfel, 1979). Social identity theory suggests that inter-group behaviors are based on perceived group status differences. Thus, if someone identifies as being part of the same group as someone else (in the case of this research – autistic people with other autistic people, or non-autistic with other non-autistic people) they may be more motivated to achieve positive results (i.e., high self-rated rapport) (Tajfel et al., 1979). In contrast, perceiving someone as being of a different group to you (in the case of this study where diagnostic status was known within mixed pairs), participants may be less motivated to have positive interactions and high self-rated rapport. Though the effect of neurotype group identity on social behavior has not been explored, when neurotypical children are assigned to different arbitrary groups (e.g., green team, blue team), they show reduced imitation of those in their outgroup, just as autistic children show reduced imitation of neurotypical children (van Schaik and Hunnius, 2016). This presents the possibility that reduced social engagement exhibited by some autistic people may be explained by a lack of identification with people from other groups (i.e., non-autistic people).

A further finding is that autistic pairs’ self-rated rapport was significantly lower than non-autistic pairs self-rated rapport. There are several reasons why this may be the case. First, autistic pairs may experience lower rapport than non-autistic pairs due to differences in processing social information. Interpersonal interactions are a rich source of social information, and it is possible autistic individuals may be placing greater emphasis on some information more than others, or have their rapport limited by the volume of interactional processing going on (Murray et al., 2005). Second, due to well-documented autistic differences in social cognition (e.g., Sasson et al., 2020) autistic people may underestimate their rapport due to negative self-perception of their social skills (Hull et al., 2017) or lower self-perceived social competence (Jamison and Schuttler, 2015). Poor self-perception may also be the consequence of having a history of negative social interactions with individuals. Future research could ask autistic individuals to assess their overall level of social competence to see if this predicts self-rated rapport on a specific dyadic interaction. Third, autistic people could make rapport judgments on dimensions not assessed by the scale used in this study. Autistic people may have a distinctive way of interacting and building rapport with others (Heasman and Gillespie, 2019a), and may make social judgments using non-traditional criteria (Morrison et al., 2020), and thus their self-rated rapport may not be well assessed by the dimensions included in this scale. Finally, autistic people may be less impacted by social desirability bias than non-autistic people (Kirchner et al., 2012), who may inflate their self-rated rapport scores to be viewed positively by the experimenter (Krumpal, 2013).

Interestingly, although Study 2 replicated the finding of reduced rapport in mixed neurotype pairs, it showed that observer-ratings of rapport indicated the opposite pattern to self-ratings in same neurotype pairs: autistic pairs were viewed as having higher interactional rapport than non-autistic pairs or mixed pairs, by both autistic and non-autistic observers. Whilst the finding of poorer rapport ratings in the mixed dyad groups as in Study 1 is again consistent with the Double Empathy account of autism, the finding of even higher ratings in the autistic pairs than non-autistic pairs is surprising. In this study, observers were blind to the neurotype of those in the videos although the participants themselves knew the diagnosis of the partner they were interacting with. One possible explanation for greater perceived rapport amongst autistic pairs could be that they immediately had something in common with the other individual (i.e., a diagnosis of autism) which may have helped them feel more at ease with one another from the start. Research showing individuals who have similar life experiences have greater social connection than those with different lived experiences supports this idea (Reagans, 2011). Although in Study 1 autistic pairs were also privy to their partners’ diagnosis status, the lower rapport ratings in the autism pairs (in relation to non-autistic pairs) may have been due to higher self-ratings in the non-autistic group. Our exploratory analysis comparing self and other ratings of rapport (Figure 4) offers support for this interpretation.

As Study 2 involves observer ratings of rapport it is important to consider the findings in relation to the broader literature on person perception. Autistic people are perceived as being more awkward and less socially warm than non-autistic people (DeBrabander et al., 2019; Morrison et al., 2020), and being difficult to read is related to being perceived unfavorably by observers (Alkhaldi et al., 2019). In Study 2, rather than asking observers to rate the characteristics of individuals, observers rated the interpersonal rapport between two people sharing an interaction. Our findings contrast somewhat with previous findings of negative perceptions of autistic individuals, and it may be that interactions offer a different perspective. As observer ratings of rapport are enhanced by stable (compared to unstable) interpersonal coordination (Miles et al., 2009), it could be that pairs of the same neurotype have similar interpersonal styles, which translate into high rapport. Autistic interactions may follow a distinctive and unconventional pattern which function to effectively facilitate mutual understanding (Heasman and Gillespie, 2019a), and it is interesting that both autistic and non-autistic viewers rate autistic pairs as having high interactional rapport using our five dimensional measure. Future work may look to identify specific verbal and non-verbal markers of interactional rapport in autistic and non-autistic interactions. While the current study illustrates that there are differences in rapport, more detailed coding of interactions may begin to explore *why* rapport is better for autistic and non-autistic people. As approaches to studying autism are framed by non-autistic definitions of being social (Heasman and Gillespie, 2019a), and autistic people have a divergent neurotype, which often makes their mode of social communication different (Kapp et al., 2013), it is essential that any future coding schemes are co-designed with autistic people to be sensitive to and incorporate autistic social behaviors.

This study does have limitations, which could be addressed by future research in this area. First, as Studies 1 and 2 have some differences in design, we are restricted in the comparisons that we can draw between the two, and in how far we can contrast self-rated and observer-rated rapport. In Study 1, the interaction was more goal-oriented, whereas Study 2 was purely conversational. However, a similar pattern of findings across both studies does suggest a robust effect in different contexts which warrants future research. Second, though fully powered to detect moderate effects, the sample size was relatively modest, and only a small number of videos were used in Study 2. Future replications should use a range of videos representing a range of ages, genders and ethnicities to ensure that they are representative of the wider community.

Third, these studies did not use a standardized measure of rapport, as a measure that was appropriate to use for both self and observer rated rapport with adults who did not know each other could not be identified, and in addition, no rapport measures have been validated for autistic respondents. Our measure assessed core rapport domains identified in Tickle-Degnen and Rosenthal (1990) empirical and theoretical work on rapport, and creating a bespoke self-rating measure including these core domains is not atypical in rapport research (e.g., Bernieri et al., 1996; Lakin and Chartrand, 2003). However we cannot fully ensure the validity of the rapport measure used. If future work pursues this line of enquiry, a measure of rapport should be developed and validated for use with neurodiverse samples.

Fourth, participants in Study 1 and those who were filmed to create the stimuli videos for Study 2 were aware of the diagnostic status of the person with whom they were interacting, which could have affected their behavior and perceptions of rapport. As participants were aware of the diagnostic status of their partner, it is possible that both autistic and non-autistic people may have experienced higher rapport within single neurotype pairs because of perceived similarity or familiarity with their interlocutor. Autistic people may feel more comfortable with other autistic people (Crompton et al., 2020a), and non-autistic people may feel more comfortable with other non-autistic people (Cage and Burton, 2019; DeBrabander et al., 2019) and this may be enhanced by being aware of the diagnostic status of the other person in the interaction. Being aware of the diagnostic status of the person with whom they were interacting may have changed participants’ behavior, however, previous research has shown that when non-autistic people know that they are interacting with an autistic person, they attempt to behave in a helpful way (Heasman and Gillespie, 2019b), and sharing diagnostic information results in greater acceptance of autistic people (Sasson and Morrison, 2019). As such, it may be hypothesized that there may be an even larger effect on rapport between mixed and single neurotype pairs if participants were blind to the diagnostic status of their partner. Although in some contexts diagnostic status may be known between individuals (e.g., peer-support groups, educational setting), at other times it may be unknown (e.g., asking a shop assistant for help). Therefore, it will be important for future research to replicate the study with participants blind to the diagnostic status of their interaction partner.

Finally, the sample may not be representative of the wider autistic community, as all participants had an IQ within a normal range, and the sample had a large proportion of female participants. As autistic males are less likely to camouflage (Hull et al., 2020), this may impact rapport, though aligning with non-autistic expectations of what autism is may result in even lower rapport in the mixed pairs.

These findings suggest that autistic difficulties in building rapport are not a deficit within an autistic individual, and instead arise within interactions with non-autistic people. Further research exploring social difficulties within and between autistic and non-autistic people could have a significant impact on our theoretical and clinical understanding of autism based on a Double Empathy framework. Specifically, our findings challenge current diagnostic criteria and theoretical framing of autism. The finding that rapport is improved between autistic people strengthens calls for peer support for autistic people (Iemmi, 2017; Crane et al., 2020), particularly since a sense of belonging is a protective factor against suicide (Pelton and Cassidy, 2017). In an educational context, these findings challenge peer mediated support practices which specifically involve pairing autistic children with non-autistic peers who are meant to act as social “role models” (Chang and Locke, 2016). In light of the current findings one should reconsider the goal of this form of peer-mediated practice, and perhaps instead emphasize the mutual benefits of interpersonal interactions between mixed neurotypes in learning about diversity in communication. Future research is needed to identify and examine the specific behaviors that facilitate rapport between autistic people, which may in turn improve interactions between people of different neurotypes.

## Data Availability Statement

The raw data supporting the conclusions of this article will be made available by the authors, without undue reservation.

## Ethics Statement

The studies involving human participants were reviewed and approved by the University of Edinburgh Psychology Research Ethics Committee, the University of Nottingham (Psychology) Research Ethics Committee, and the University of Durham (Education) Research Committee. The patients/participants provided their written informed consent to participate in this study.

## Author Contributions

CC: study design, data collection, data analysis, and led manuscript writing. MS and HA: data collection and revised manuscript. SF-W: project creative and scientific design, data analysis, and revised manuscript. EF: project creative and scientific design, revised manuscript. DR: project creative and scientific design, data analysis, and co-wrote the manuscript. All authors contributed to the article and approved the submitted version.

## Conflict of Interest

The authors declare that the research was conducted in the absence of any commercial or financial relationships that could be construed as a potential conflict of interest.
