# Supplementary material for: Neurotype-Matching, but Not Being Autistic, Influences Self and Observer Ratings of Interpersonal Rapport
Source: Front Psychol. 2020 Oct 23;11:586171. doi: 10.3389/fpsyg.2020.586171 (PMC7645034; doi:10.3389/fpsyg.2020.586171)
Supplement: Supplementary file 1 [file Data_Sheet_1.PDF]

Supplementary information

Calculating sum mean rapport scores

Pair mean rapport scores for the three diffusion chain tasks were first compared to examine whether there was any significant difference in pair mean rapport between the tasks. A repeated measures ANOVA determined that pair mean rapport scores differed between the three tasks ($F(2,180) = 7.76$, $p = 0.0005$), and the three groups ($F(2, 180) = 39.75$, $p = 0.00005$) but that there was no significant interaction between task and group ($F(4,180) = 0.28$, $p = 0.89$. As there was no interaction between the task and group, a summed mean was used in subsequent analyses, calculated by summing the paired mean rapport scores for each of the three tasks.

[Supplementary Figure 1]

Figure 1: pair mean rapport scores for the three tasks in autistic, non-autistic, and mixed groups.

Table 1: Descriptive statistics (Mean (Standard Deviation) on Study 1 demographics by neurotype. Comparisons by independent t-test and Fisher's exact test comparisons.

	Non-autistic	Autistic	Comparison
Age	36.31 (13.00)	37.36 (12.59)	t (69,93) = 0.35, p = 0.73
Gender	29F, 7M	28F, 5M, 3 NB	Fisher's exact test p = 0.24
Years of Education	17.74 (1.81)	17.19 (2.45)	t(64.44) = -1.07, p = 0.29
IQ (WASI-II)	115.86 (10.71)	115.64 (17.03)	t (58.92) = -0.67, p = 0.95
Autism Quotient	13.67 (5.87)	36.78 (6.60)	t (69.07) = 15.69, p < 0.0001
Age of Diagnosis	NA	30.66 (11.81)	NA

[a] Wechsler Abbreviate Scale of Intelligence -II [b] Non-binary

Table 2: Descriptive statistics and group comparisons (Mean (Standard Deviation) for participants featured in Study 2 video stimuli. No formal comparisons of these participants has been carried out due to the small sample size.

	Non-autistic (n = 6)	Autistic (n = 6)	Non-autistic in mixed pair (n =3)	Autistic in mixed pair (n=3)
Age	45.67 (12.86)	47.50 (16.73)	38.67 (13.61)	41.00 (8.66)
Gender	6F	1M, 4F, 1NB	3F	2F, 1 M
AQ score	10.67 (4.76)	32.67(6.74)	12.00 (5.56)	38.67 (5.13)
