# Supplementary figures and images for: Neurotype-Matching, but Not Being Autistic, Influences Self and Observer Ratings of Interpersonal Rapport
Source: Front Psychol. 2020 Oct 23;11:586171. doi: 10.3389/fpsyg.2020.586171 (PMC7645034; doi:10.3389/fpsyg.2020.586171)

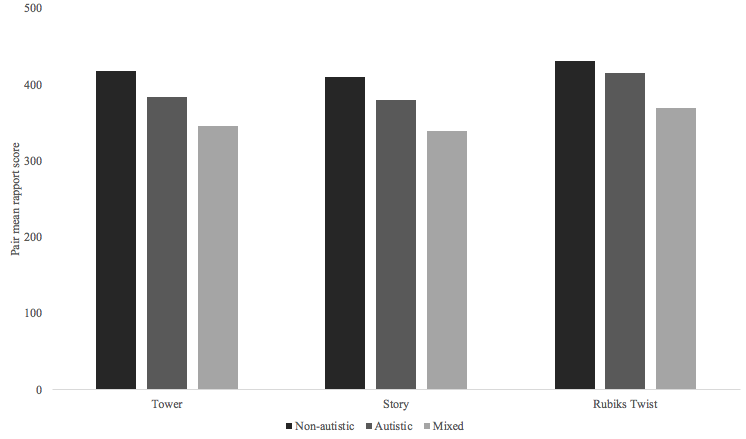

Supplement: Supplementary file 2 [file Image_1.TIFF]
